# Can nasal acetylsalicylic acid challenge predict the severity of non-steroidal anti-inflammatory drugs (NSAIDs)-exacerbated respiratory disease (N-ERD)? 

**DOI:** 10.5414/ALX01996E 

**Published:** 2020-12-23

**Authors:** Ulrike Förster-Ruhrmann, Anne-Kristin Tietz, Jonghui Kim, Uta Liebers, Agnieszka J. Szczepek, Heidi Olze

**Affiliations:** 1 CVK Department of Otorhinolaryngology, Head and Neck Surgery, Charité – Universitätsmedizin Berlin, Corporate Member of Freie Universität Berlin, Humboldt-Universität zu Berlin, and Berlin Institute of Health, Berlin,; 2 Department of Pulmonology, Charité – Universitätsmedizin Berlin, Corporate Member of Freie Universität Berlin, Humboldt-Universität zu Berlin, and Berlin Institute of Health, Berlin, and; 3 CCM Department of Otorhinolaryngology, Head and Neck Surgery, Charité – Universitätsmedizin Berlin, Corporate Member of Freie Universität Berlin, Humboldt-Universität zu Berlin, and Berlin Institute of Health, Berlin, Germany

**Keywords:** nasal polyps, CRSwNP, NSAIDs hypersensitivity, N-ERD, nasal ASA challenge, asthma, ACT-score

## Abstract

Background: Non-steroidal anti-inflammatory drugs (NSAIDs)-exacerbated respiratory disease (N-ERD) complicates the clinical course of chronic rhinosinusitis with nasal polyps (CRSwNP) and severe asthma. We aimed to determine the detection rate of NERD in patients with CRSwNP, asthma, and history of NSAID intolerance using nasal challenge with acetylsalicylic acid (ASA) and the relationship between the severities of response to ASA challenges and the grade of N-ERD. Materials and methods: Three groups of patients were included: CRSwNP with asthma and clinical history of analgesics intolerance (CRSwNP-AAI n = 18), CRSwNP with asthma but without a clinical history of analgesics intolerance (CRSwNP-A n = 20), and CRSwNP without asthma or analgesics intolerance (n = 18). All subjects were challenged nasally with 16 mg ASA and monitored with active anterior rhinomanometry. Rhinological (nasal polyp score), pulmonary (spirometry, Asthma Control Test (ACT), and asthma treatment), and psychometric questionnaire scores were recorded and correlated with rhinomanometric data following nasal challenges (flow depressions and symptom scores). Results: Nasal ASA challenge detected N-ERD in 96.7% of CRSwNP-AAI patients and 45% of CRSwNP-A patients. No N-ERD was seen in the CRSwNP group. The control grade of asthma measured with ACT scores was significantly lower in the groups CRSwNP-AAI (MV 18.22) and CRSwNP-A (MV 19.75) when compared to the CRSwNP group (MV 24.39) (p = 0.000). In the CRSwNP-AAI group, 11 patients had uncontrolled asthma (61%), and in the CRSwNP-A group, 9 patients had uncontrolled asthma (45%). No correlation was found between rhinology and pulmonary parameters, nasal symptoms, and the severity of nasal ASA challenges. Specific reactions were detectable under the therapy of prednisolone and omalizumab. Conclusion: N-ERD might not always be detected by screening a patient’s medical history. Nasal ASA challenges are recommended in patients with CRSwNP and asthma. The nasal challenge with ASA positively confirms the N-ERD diagnosis. Moreover, N-ERD is a differential diagnosis in patients with severe asthma with the need for prednisolone or omalizumab therapy. The severity of the reaction to the ASA challenge in controlled and uncontrolled asthma patients is independent of the grade of N-ERD.

**German version published in Allergologie, Vol. 41, Nr. 12/2018, pp. 551-559**

## Introduction 

The symptoms of non-steroidal anti-inflammatory drugs (NSAIDs)-exacerbated respiratory disease (N-ERD) include hypersensitivity to NSAIDs, asthma, and/or chronic rhinosinusitis with nasal polyps (CRSwNP). Patients with CRSwNP and N-ERD frequently undergo nasal surgeries [1]. Furthermore, in individuals with N-ERD, the progression of asthmatic disease occurs often, and oral corticosteroids are commonly administered [2, 3]. Bochenek et al. [4] have examined asthma patients with N-ERD and detected uncontrolled asthma in 45% of cases with an Asthma Control Test (ACT) < 20. In a consensus report, Lötvall et al. [5] proposed that acetylasicylic acid-sensitive asthma is a separate endotype of asthma. 

Inflammatory endotypes of chronic rhinosinusitis have recently been described by Tomassen et al. [6] based on cluster analysis of biomarkers. The inflammatory process occurring in N-ERD patients is characterized by the overproduction of cysteinyl leukotrienes and marked eosinophilia in the nasal polyps and bronchoalveolar lavage. In addition, cytokines like IL-4, IL-5, and IFN-γ amplify the inflammatory course of disease [7, 8]. In our own studies, we have demonstrated an eicosanoid imbalance in peripheral blood, and the grade of imbalance correlated with clinical symptoms such as smell impairment, nasal congestion, and rhinorrhea [9]. 

For the clinical assessment of NSAIDs hypersensitivity, challenge with ASA via either oral, nasal, or bronchial routes are used. Recommendations for the oral, nasal, and bronchial ASA challenge procedures have been recently published in the European EAACI/GA2LEN guidelines [10]. The guidelines report 89% sensitivity and 93% specificity for an oral ASA challenge, followed by 87% sensitivity and 95.7% specificity for a nasal challenge, and lastly 77% sensitivity and 93% specificity for a bronchial challenge. 

To date, little is known about the association between the grade of N-ERD and the severity of the reaction to nasal ASA challenge (a rhinomanometric measurement of flow depression and symptom scores). If the results of the nasal challenge are negative, then oral tests are recommended. However, due to possible adverse reactions, oral and bronchial ASA challenges require hospital admission, whereas the nasal ASA provocation is relatively safe and can be performed in an outpatient department. The EU guidelines recommend using nasal ASA challenges in patients with severe asthma, but to date, there is a lack of systematic studies regarding this topic [11, 12]. Our present study was designed to determine the detection rate of N-ERD using nasal challenge with ASA in patients with CRSwNP, asthma, and uncertain or certain history of a hypersensitive reaction to NSAIDs. Furthermore, we wanted to determine the association between clinical parameters (nasal, pulmonary parameters, and nasal symptoms of patients) and the outcome of rhinomanometric measurements (flow depressions and symptom scores). 

## Materials and methods 

### Patients 

The patients admitted to the ENT department were included in the study between 2014 and 2015 upon signing written consent. This prospective study strictly followed the declaration of Helsinki and obtained the permission of the local Ethics Committee. Patients with CRS and nasal polyps as defined by EPOS criteria (European Position Paper on Rhinosinusitis and Nasal Polyps) [13] and with asthma as defined by GINA criteria (Global Initiative for Asthma) [14] were included in the study. 

**Following inclusion criteria were applied **

1. CRSwNP-AAI: chronic rhinosinusitis with nasal polyps, asthma, history of analgesics intolerance with respiratory symptoms (e.g., rhinitis, asthma);
2. CRSwNP-A: chronic rhinosinusitis with nasal polyps, asthma, no history of analgesics intolerance;
3. CRSwNP: patients with CRSwNP, no asthma, no history of analgesics intolerance.

**General exclusion criteria**

Age under 18 years, acute rhinosinusitis or asthma exacerbation, bilateral CRSwNP with total obstruction of the nose. 

**Patients**

CRSwNP-AAI: 18 patients, 6 men, 12 women, age mean value (MV) 46.6 years; standard deviation (SD) 15.0; 

- CRSwNP-A: 20 patients, 9 men, 11 women, age (MV) 50.3 years; SD 11.2; 
- CRSwNP: 18 persons, 13 men, 5 women, age (MV) 42.4 years; SD 14.5; 

**ASA intake**

CRSwNP-A: no ASA intake in 11 patients (55%), no intolerance reactions in 9 patients despite ASA intake (45%); 

CRSwNP: no ASA intake in 10 patients (56%), no intolerance reactions in 8 patients despite ASA intake (44%). 

### Methods 

**Parameters evaluated**

*Rhinological parameters*

The number of nasal sinus surgeries was noted as a score. The nasal polyps were evaluated using the Davos endoscopy score [15], and the smell was tested using the Burghart Sniffin’ Sticks olfaction test (Burghart Messtechnik GmbH, Wedel, Germany). 

*Pulmonary parameters*

Spirometry was performed with forced expiratory volume 1 (FEV1). Asthma was assessed using the Asthma Control Test (ACT). A score of 25 – 20 points indicated controlled asthma, whereas a score < 20 indicated uncontrolled asthma. Additionally, the severity of asthma was assessed based on current asthma medication, according to the criteria of the Global Initiative for Asthma (GINA) [14].

*Questionnaires of CRS symptoms and general quality of life*

The severity of CRS symptoms such as nasal obstruction, anterior/posterior rhinorrhea, olfactory impairment, and facial pain were scored using a visual analog scale (VAS) [13]. The general quality of life was measured with the Short Form-36 Health Survey (SF-36).

*Restriction time of medical drugs before ASA challenge*

Restriction time for nasal α-adrenergic drugs was 24 hours and for antihistamines 3 days. Before the nasal challenge, montelukast was postponed for at least 1 week, omalizumab for 4 weeks, whereas prednisolone (inhaled and oral) was left unaltered to maintain asthma control. 

**Nasal challenge**

The nasal challenge was performed according to the German guidelines [16]. The nasal airflow and resistance were measured using active anterior rhinomanometry (BD/CareFusion Germany GmbH, Hoechberg, Germany). All patients were challenged in an outpatient ward. The nasal flow (cm^3^/s) was measured on both sides, and the wider side was used for the challenge. 

First, 80 µL placebo (isotone saline) was applied to the inferior lateral nasal mucosa of the test side. Next, 80 µL of lysine-aspirin solution (L-ASA, Bayer Health Care Pharmaceuticals, Leverkusen, Germany) containing 16 mg of ASA was applied to the test side. After the ASA application, flow depressions were examined, and symptoms were measured every 10 minutes for a maximum of 3 hours, or until the response occurred. The symptoms were scored semi-quantitatively in agreement with the German guidelines (Table 1) [16]. N-ERD was diagnosed in case of a positive outcome of the nasal ASA challenge. 

**Statistics**

The Mann-Whitney U test and the Spearman’s rank correlation (p < 0.05) were used. The significance level was set to p < 0.05. 

## Results 

### Results of the nasal challenge with ASA 

In the CRSwNP-AAI group of patients with a previous clinical history of analgesics intolerance, 17 of 18 patients reacted positively to the nasal ASA challenge (94.4%). In the CRSwNP-A group of patients without a clinical history of analgesics intolerance, 9 (45.0%) patients had a positive reaction. In the CRSwNP group, there were no positive reactions. 

**Figure 1:**
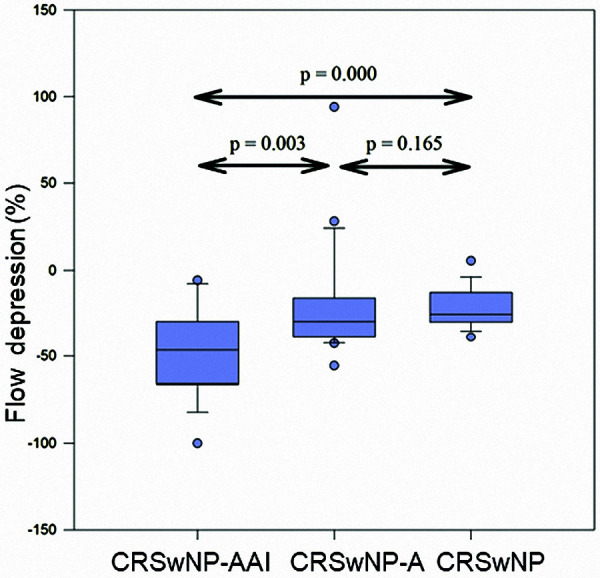
Between-group comparison of maximal flow depression (16 mg ASA).

**Figure 2:**
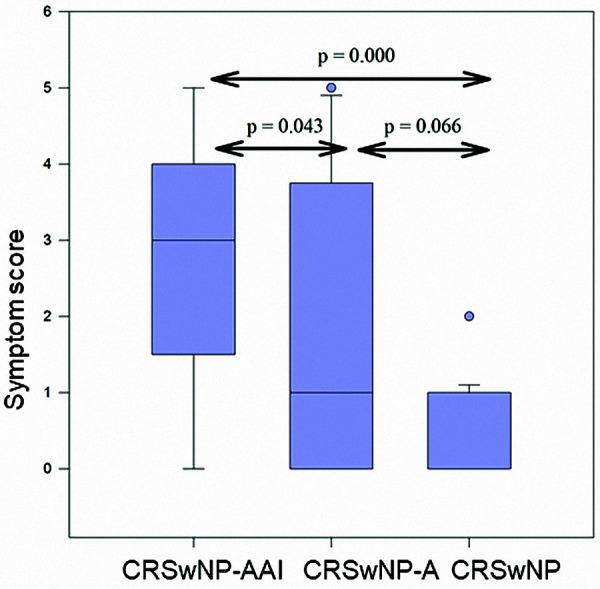
Between-group comparison of symptom score (16 mg ASA).

CRSwNP-AAI patients had significantly greater flow depression (MV –47.6% (–7.9 to –100%)) (Figure 1) and the highest symptom score (MV 2.7 (0 – 5)) (Figure 2) as compared to CRSwNP-A (flow depression MV –21.1% (–55.6 to –10.2%), p = 0.003; symptom score MV 1.6 (0 – 5), p = 0.043) and CRSwNP (flow depression MV –21.4% (–4.7 to -38.9%), p = 0.000; symptom score MV 0.4 (0 – 2), p = 0.000). The mean time until the occurrence of a positive reaction was 43.3 minutes (10 – 150). 

### Increased ASA reactions after nasal ASA challenge 

Following the nasal ASA challenge, 4 (22.2%) of the CRSwNP-AAI patients developed mild pulmonary symptoms. Following inhalation with salbutamol, the side effects disappeared rapidly. In addition, 2 of these patients received 250 mg prednisolone intravenously. 

One patient developed conjunctivitis, and 1 patient erythema on the face, but no treatment was necessary in these cases. 

### Group comparison regarding rhinological and respiratory parameters 

The CRSwNP-AAI group had significantly more nasal polyps (MV 1.72) than the CRSwNP group (MV 0.72, p = 0.033), but there were no significant differences when compared to the number of nasal polyps in the CRSwNP-A group (MV 1.25) (Table 2). The CRSwNP-AAI group had significantly greater olfactory impairment (MV 4.89) than the CRSwNP group (MV 8.22, p = 0.029). Between-group comparison of FEV1 values revealed no significant differences (CRSwNP-AAI MV 86.7% (38 – 116%); CRSwNP-A MV 90.1 (68.5 – 117%), p = 0.76) (Table 2). The control grade of asthma measured with ACT scores was significantly lower in the groups CRSwNP-AAI (MV 18.22) and CRSwNP-A (MV 19.75) when compared to the CRSwNP group (MV 24.39) (p = 0.000). Specifically, 11 patients in the CRSwNP-AAI group (61%) and 9 patients in the CRSwNP-A group (45%) had uncontrolled asthma. Table 3 demonstrates the asthma medication and the asthma treatment steps according to the GINA guidelines of the asthma patient groups. Omalizumab therapy was given in 4 cases in the CRSwNP-AAI group (22.2%) and in 2 cases in the CRSwNP-A group (15%) (Table 3). 45% of the CRSwNP-AAI group and 15% of CRSwNP-A group had the most severe treatment step 5 to control asthma (daily oral prednisolone MV 14 mg in CRSwNP-AAI group and MV 15 mg in CRSwNP-A group). All patients with prednisolone and/or omalizumab in the CRSwNP-AAI group, and 1 of 2 cases in the CRSwNP-A group showed specific reactions after ASA challenge. No statistically significant differences for the GINA score could be shown between the asthma patient groups (CRSwNP-AAI MV 3.9; CRSwNP-A MV 3.2; p = 0.034). 

### Group comparison regarding VAS scores and SF-36 

CRSwNP-AAI patients (MV 70.50) had significantly higher VAS scores indicating the severity of disease as compared to the CRSwNP patients (MV 47.17, p = 0.017). Furthermore, the CRSwNP-A group had higher VAS scores indicating the severity of disease (MV 70.90) and smell impairment (MV 73.15) than the CRSwNP group (MV 47.17, p = 0.014; MV 48.50, p = 0.043), but not the CRSwNP-AAI group. The general quality of life (physical summary score) measured by the SF-36 questionnaire indicated a worse quality of life in the CRSwNP-AAI patients (MV 37.58) as compared to the CRSwNP patients (MV 48.13, p = 0.002) (Table 4). 

### Correlation of rhinomanometric parameters (flow depression and symptom scores) with rhinological, respiratory parameters, and questionnaires of CRS symptoms and quality of life 

To determine the possible correlation between the severity of nasal ASA challenge and the grade of disease, “flow depression” and “symptom scores” of nasal challenges were computed with the following parameters: 

- age 
- nasal polyp score, number of nasal sinus surgeries, olfactory test 
- pulmonary parameters (FEV1 value, ACT score, asthma medication score) 
- scores obtained from VAS and SF-36. 

The correlations were performed in two groups. The first group consisted of patients with positive nasal reactions after ASA provocation, and the second group consisted of the patients with adverse nasal responses. The only positive significant correlation was identified between the flow depression and the SF-36 score in patients with positive nasal reactions after ASA challenge. 

## Discussion 

In this study, we determined the detection rate of N-ERD using nasal challenge with ASA in patients with CRSwNP, asthma, and uncertain or certain history of hypersensitive reaction to NSAIDs. In the case of an isolated CRSwNP without asthma, there is no recommendation for ASA provocation. 

Our important new finding is that the degree of rhinomanometric reactions induced by a nasal ASA challenge does not depend on the severity of the disease. Lastly, we demonstrate that patients with uncontrolled asthma can be nasally challenged with ASA, which agrees with only a few existing studies on that topic [11, 12]. 

The procedure of nasal provocation test using 16 mg of ASA in the present study confirmed N-ERD diagnosis in 94.4% of patients with CRSwNP, asthma, and suspected ASA hypersensitivity-specific reactions. In contrast, other studies report a sensitivity of 38 – 87% during similar testing [17, 18]. Our analysis of the CRSwNP-AAI patients confirms the effectiveness and accuracy of the nasal challenge with ASA. 

Szczeklik et al. [19] performed a multicenter study covering 500 ASA-hypersensitive patients with asthma. Among them, 15% were not aware of their ASA hypersensitivity, which was revealed by ASA challenges. Similar results were reported by Vaidyanathan et al., [20] who studied 75 CRSwNP patients and after the nasal challenge with 16 mg of ASA found that although only 31% had a positive history of ASA hypersensitivity, 51% reacted positively to ASA nasal challenge. 

Miller et al. [21] obtained similar results when using nasal challenge with a maximum of 40 mg of ASA. Of the 150 patients included in that study, 79% had CRSwNP, 88% asthma, and 57% had a positive history of ASA hypersensitivity. Also here, more patients than expected (67%) reacted positively to the nasal ASA challenge. In the present study, patients with CRSwNP and asthma without a positive history of NSAIDs hypersensitivity were assigned to the group CRSwNP-A. Of these, 9 patients (45%) reacted positively to the nasal provocation test. A possible explanation of this observation can be the avoidance of NSAIDs intake, as asthma patients are often advised by their physicians to avoid ASA. 

Nizankowsla-Mogilnicka et al. [10] recommended in the EU guidelines of nasal NSAIDs challenges a maximum daily dosage of oral 10 mg prednisolone before the oral ASA challenges. In our study, we have not modulated the intake of oral prednisolone to maintain asthma control; however, despite a higher average dosage of prednisolone (MV 14.4 mg CRSwNP-AAI), specific ASA reactions were still detectable. Furthermore, specific ASA reactions were also seen under omalizumab therapy. 

We observed mild pulmonary reactions affecting 22.2% of the patients with CRSwNP-AAI (8.9% of the total sample). This frequency is similar to that described earlier, ranging between 12.5 and 21% [21, 22]. The reasons for pulmonary reactions may be micro-aspirations after the nasal administration of ASA. 

Nasal ASA challenges were also carried out by other research groups in patients with severe asthma, but there was no systematic investigation of patients with severe asthma [11, 12]. In our analysis, the FEV1 values with a minimum of 38% in the CRSwNP-AAI group and 68% in the CRSwNP-A group did not differ between the groups. 

Similarly, no significant differences in the FEV1 values between 20 ASA-intolerant and 14 ASA-tolerant asthma patients were previously found [23]. This is in contrast with the results of Mascia et al., [24] who detected significantly worse FEV1 values in ASA-intolerant asthmatics. The ACT scores of our patients belonging to the CRSwNP-AAI and CRSwNP-A groups were significantly reduced in contrast to the scores of the CRSwNP group, and without significant differences between the asthma groups. No significant differences were previously identified when comparing ACT scores of ASA-intolerant and ASA-tolerant subjects with asthma [25]. Interestingly, the majority of our asthma patients had uncontrolled asthma despite pulmonary treatment (CRSwNP-AAI 61% and CRSwNP-A 45%), whereas among 201 N-ERD patients described earlier, only 45% had uncontrolled asthma [4]. This strongly implies the need to identify patients with NERD and improve the therapy for asthma. 

In contrast to severe asthma, the nasal polyp score observed in the present study was low, which may be due to the preselection of the patients with frequent pulmonary referrals to the university outpatient department. 

There were no significant differences regarding nasal symptoms measured by VAS, which is similar to our earlier data [9]. The scores indicating the quality of life (SF-36) revealed almost no significant differences between the patient groups. The CRSwNP-AAI group reported significantly worse values for the quality of life (physical summary score) than the CRSwNP group. Alobid et al. [26] found similar lower quality of life in N-ERD patients than in patients without asthma. 

The results of the present study suggest that the degree of flow depressions and symptom scores of nasal challenges do not depend on the severity of N-ERD in patients with controlled and uncontrolled asthma. Similarly to our findings, Vaidyanathan et al. [20] indicated that positive nasal ASA challenge is independent of the severity of CRSwNP with and without asthma. Nevertheless, correlations of rhinological and pulmonary parameters with the rhinomanometric measurement parameters were not performed there. Williams et al. [27] detected no connection between the severity of historical asthma reactions due to the severity of asthma reactions following oral ASA challenge. However, in contrast to our investigation, Williams et al. [27] and Vaidyanathan et al. [20] did not include ASA-intolerant patients with severe uncontrolled asthma in the studied sample. 

## Conclusion 

Nasal ASA provocations are useful in CRSwNP patients with asthma and without previously known analgesics intolerance, since the analgesics intoleranceis often not reported in medical history. 

Nasal ASA challenges effectively confirm the N-ERD diagnosis. Specific ASA reactions are also detectable under therapy with oral corticosteroids or omalizumab. The outcome of nasal ASA provocations is independent of rhinological, pulmonary parameters, or the severity of subjective patient complaints, which applies to both patients with controlled and uncontrolled asthma. 

## Funding 

None. 

## Conflict of interest 

UFR, AKT, JK, UL, AJS, and HO declare no conflict of interest. 

**Table 1.** Criteria of a positive nasal challenge according to the German guidelines of Riechelmann et al. [16].

1. Flow reduction > 40% on the tested side	
2. *or* Flow reduction > 20% on the tested side and symptom score > 2	
3. *or* Symptom score > 3;	*Symptom score:*
	Secretion: no/ mild/ severe (0; 1; 2 points);
	Distant symptoms: eye secretion and/ or oral itching and/ or ear itching (1 point); conjunctivitis and/ or chemosis and/or urticaria and/or cough and/ or dyspnea (2 points);
	Irritation: ≤ 2/ 3 – 5/ > 5 sneezing (0; 1; 2 points);

**Table 2.** Rhinological and pulmonary parameters of patients groups and between-group comparison.

	CRSwNP-AAI	CRSwNP-A	CRSwNP	CRSwNP-AAI/ CRSwNP-A	CRSwNP-AAI/ CRSwNP	CRSwNP-A/ CRSWNP
**Rhinological parameters**						
Nasal polyp-score	MV 1.72 SD 1.74	MV 1.25 SD 1.37	MV 0.72 SD 1.36	p = 0.414	p = 0.033	p = 0.142
Number of nasal operations	MV 2.56 SD 1.62	MV 1.85 SD 1.23	MV 2.39 SD 2.28	p = 0.155	p = 0.444	p = 0.548
Smell test	MV 4.89 SD 4.92	MV 6.50 SD 5.33	MV 8.22 SD 3.77	p = 0.401	p = 0.029	p = 0.268
**Pulmonal parameters**						
FEV1 (%)	MV 86.72 SD 18.26	MV 90.09 SD 14.96	MV 99.28 SD 11.53	p = 0.760	p = 0.062	p = 0.056
ACT-score	MV 18.22 SD 4.73	MV 19.75 SD 4.30	MV 24.39 SD 1.14	p = 0.223	p = 0.000	p = 0.000

CRSwNP-AAI = chronic rhinosinusitis with NP; asthma and clinical history of analgesics intolerance; CRSwNP-A = chronic rhinosinusitis with NP; asthma without a clinical history of analgesics intolerance; CRSwNP = chronic rhinosinusitis with NP without asthma or analgesics intolerance; FEV1 = forced expiratory volumen in 1 second; ACT = asthma-Control-Test; MV = median value; NP = nasal polyps; SD = standard deviation.

**Table 3.** Asthma medication.

	CRSwNP-AAI	CRSwNP-A
Number	18	20
ICS low dose	6 (33%)	10 (50%)
ICS moderate dose	5 (29%)	2 (20%)
ICS high dose	4 (22%)	4 (20%)
OCS Daily dose	5 (27%) MV 14.4 mg (5 – 20)	1 (5%) 15 mg
Omalizumab	4 (22%)	2 (10%)
Combination of OCS/Omalizumab	1 (6%)	1 (5%)
GINA-score	3.9 (1 – 5)	3.2 (1 – 5)

ICS = inhaled corticosteroid; OCS = oral corticosteroid; MV = median value.

**Table 4.** Scores obtained within the tested groups with the use of VAS and SF-36 and between-group comparison.

	CRSwNP-AAI	CRSwNP-A	CRSwNP	CRSwNP-AAI/ CRSwNP-A	CRSwNP-AAI/ CRSwNP	CRSwNP-A/ CRSWNP
**VAS scores**						
Severity of disease	MV 70.50 SD 25.19	MV 70.90 SD 26.82	MV 47.17 SD 31.41	p = 0.767	p = 0.017	p = 0.014
Nasal obstruction	MV 60.89 SD 31.48	MV 59.00 SD 22.72	MV 55.06 SD 33.87	p = 0.492	p = 0.821	p = 0.823
Facial pain/-pressure	MV 32.17 SD 28.29	MV 22.20 SD 24.17	MV 15.78 SD 28.00	p = 0.463	p = 0.105	p = 0.092
Rhinorrhoea anterior	MV 42.00 SD 28.18	MV 46.35 SD 31.97	MV 40.56 SD 30.35	p = 0.608	p = 0.870	p = 0.539
Rhinorrhoea posterior	MV 34.89 SD 31.49	MV 44.75 SD 33.76	MV 35.50 SD 29.31	p = 0.482	p = 0.870	p = 0.510
Smell impairment	MV 58.28 SD 39.57	MV 73.15 SD 26.96	MV 48.50 SD 40.04	p = 0.3334	p = 0.418	p = 0.043
**SF-36**						
Physical summary score	MV 37.58 SD 9.66	MV 42.70 SD 9.71	MV 48.13 SD 7.59	p = 0.120	p = 0.002	p = 0.087
Mental summary score	MV 46.36 SD 9.10	MV 42.46 SD 12.78	MV 49.34 SD 9.91	p = 0.496	p = 0.372	p = 0.092

VAS = visual analog scale; SF-36 = Short Form-36; MW = median value; SD = standard deviation.
